# Active Cardboard Box with Smart Internal Lining Based on Encapsulated Essential Oils for Enhancing the Shelf Life of Fresh Mandarins

**DOI:** 10.3390/foods9050590

**Published:** 2020-05-06

**Authors:** Antonio López-Gómez, María Ros-Chumillas, Laura Buendía-Moreno, Laura Navarro-Segura, Ginés Benito Martínez-Hernández

**Affiliations:** 1 Food Safety and Refrigeration Engineering Group, Department of Agricultural Engineering, Universidad Politécnica de Cartagena, Paseo Alfonso XIII, 48, 30203 Cartagena, Spain; may.ros@upct.es (M.R.-C.); laura.buendia.moreno@gmail.com (L.B.-M.); laura.navarro@upct.es (L.N.-S.); GinesBenito.Martinez@upct.es (G.B.M.-H.); 2 Biotechnological Processes Technology and Engineering Lab, Instituto de Biotecnología Vegetal, Universidad Politécnica de Cartagena, Edif I+D+I, Campus Muralla del Mar, 30202 Cartagena, Murcia, Spain

**Keywords:** *β*-cyclodextrin, inclusion complex, carvacrol, essential oils, active packaging, citrus, quality, shelf life, decay incidence

## Abstract

Mandarins are usually sold in bulk and refrigerated in open cardboard boxes with a relatively short shelf-life (12–15 days) due to physiological and pathological disorders (rot, dehydration, internal breakdown, etc.). The influence of a controlled release of essential oils (EOs) from an active packaging (including *β*-cyclodextrin-EOs inclusion complex) was studied on the mandarin quality stability, comparing different sized cardboard trays and boxes, either non-active or active, at the pilot plant scale (experiment 1; commercialization simulation at room temperature after a previous simulation of short transportation/storage of 5 days at 8 °C). Then, the selected package was further validated at the industrial scale (experiment 2; cold storage at 8 °C up to 21 days). Among package types, the active large box (≈10 kg fruit per box) better maintained the mandarin quality, extending the shelf life from two weeks (non-active large box) to three weeks at room temperature. Particularly, the active large box highly controlled microbial growth (up to two log units), reduced weight losses (by 1.6-fold), reduced acidity, and increased soluble solids (highly appreciated in sensory analyses), while it minimized colour and controlled firmness changes after three weeks. Such trends were also observed during the validation experiment, extending the shelf life (based on sensory quality) from 14 to at least 21 days. In conclusion, the mandarin’s shelf life with this active cardboard box format was extended more than one week at 8 °C.

## 1. Introduction

Mandarin (*Citrus reticulata*) is a citrus fruit consumed worldwide with a total production of approximately 34 million tonnes in 2018 [1]. The production of mandarins (including clementines, satsumas and different hybrid mandarins) occupies the second place of total citrus fruit production followed by lemons, limes, and grapefruits [1]. Nevertheless, since a high proportion of oranges, the most cultivated citrus fruit, are used for juice extraction [2], mandarin production could thus be considered as the most cultivated citrus fruit for fresh consumption.

The high popularity of mandarins among consumers is explained by its characteristic convenience (easy to peel and eat), palatable sweetness-acidity binomial, and high content of bioactive compounds, mainly vitamin C [3,4]. The sweetness and acidity of mandarins, and citrus fruit in general, have been conventionally measured with soluble solids content (SSC) and titratable acidity (TA) determinations. This important sweetness-acidity binomial has been quantified using maturity indexes like the conventional SSC/TA index, and the BrimA (an abbreviation for “Brix minus Acids”) index [5], as recommended for citrus [6]. These maturity indexes have been highly correlated to the fruit flavour together with peel colour, which may influence the sensory acceptance highly influenced by them [6,7]. The development of citrus peel colour during maturation is associated to the coordinated carotenoid biosynthesis and chlorophyll degradation [7]. In particular, up to 94% of total carotenoid content in mandarins are present in the peel after colour beak. Specifically, *β*-cryptoxanthin and apocarotenoids are the carotenoids responsible for the distinctive orange-reddish pigmentation in mandarins [7]. The peel colour is the main attribute of mandarins determining the consumer decision to purchase them, together with size and blemish-free [8]. Several colour indexes calculated using CIE and Hunter coordinates (mainly L, a and b) have been proposed to determine the orange colour changes in citrus with a single and representative colour index. In particular, *a/b* index, which increases with increasing yellow to red colour, was proposed early for citrus due to the high correlation with the United States Department of Agriculture (USDA) colour standards [9,10]. Furthermore, ((1000 × a)/(L × b)) index was proposed for predicting the fruit colour changes during the degreening of citrus since it correlated well with the visual appreciation of peel colour changes of citrus from dark green to orange [11]. Citrus fruit flesh is not as firm as other fruits due to the characteristic presence of juice sacs. Nevertheless, citrus firmness is important since flesh firmness of citrus fruits, and mandarin in particular, highly influences their mouth feel [12].

Mandarin postharvest losses can have physiological, pathological or physical (e.g., rind wounds, bruises) origin, with weight loss and physiological disorders being the major causes of such postharvest losses [2]. Weight loss does not result only in direct quantitative losses (=economic losses), leading also to the triggering of physiological disorders like peel pitting, stem-end rind breakdown, shrivelling, collapse of the stem-end button, etc. [2,13]. Mandarins are also sensitive to chilling injuries when the storage temperature is below 5 °C, characterized by pitting and brown discolouration followed by increased susceptibility to decay. Therefore, the recommended storage temperature for mandarins is 5–8 °C [13]. Among the main pathological disorders of citrus fruits, mainly caused by filamentous fungi are stem-end rots, green mould, blue mould, grey mould, black rot, brown rot, and anthracnose [2].

Postharvest treatments conventionally used to maintain the quality of citrus and extend their shelf life are waxes and/or chemical fungicides. Nevertheless, such conventional postharvest treatments have raised important health and environmental issues, as the residue levels of such agrochemicals that are progressively more restricted by official regulations. In that sense, alternative and eco-sustainable postharvest treatments are needed, presenting essential oils (EOs) an interesting opportunity due to their high antimicrobial properties.

EOs are oily liquids extracted from plants that display a high in vitro antimicrobial activity. Carvacrol is the major component of oregano EOs with wide spectra against both gram-negative (e.g., enterobacteria) and gram-positive bacteria, and other microbial groups like moulds [14,15,16,17]. Nevertheless, in vivo effectiveness of EOs is reduced due to their evaporation and other light and oxygen degradative reactions. In that sense, higher EO concentrations are needed in vivo to reach the same effectiveness in vitro, with the consequent appearance of EO-related off-flavours [18]. Furthermore, EO mixes including the major EO components (e.g., carvacrol) together with its correspondent EOs (e.g., oregano EOs) have shown a synergistic effect on the antimicrobial activity [19]. In that sense, an encapsulated EOs mix composed of carvacrol:oregano EO:cinnamon EO (70:10:20; weight (*w*):*w*:*w*) showed a high antimicrobial effect in plant products packaged with this active package [11,20]. Nanoencapsulation can greatly reduce EOs oxidation and evaporation while ensuring a controlled release of EOs in small concentrations to the surrounding atmosphere. Cyclodextrins (CDs) (cyclic oligomers of α-d-glucopyranose with a hydrophobic cavity) can highly encapsulate EOs avoiding their oxidation, light-degradation, evaporation, etc. [15,21]. The most important CDs at the industrial level are α- and β-CDs. In particular, β-CD is highly extended due to its low cost. *β*CD is approved as a food additive in Europe (E459), USA, and Japan, with an acceptable daily intake of 5 mg kg^−1^ (body weight) day^−1^ [22].

Antimicrobial active packaging is an emerging technology that allows extending the food shelf life through a controlled release of the encapsulated antimicrobial compounds [23]. Corrugated cardboard is widely used in the European Union as an eco-sustainable packaging material for packaging of fresh fruit and vegetables. Furthermore, cardboard is highly used for mandarins under different formats (boxes, trays, flock-packs, alveoli trays, etc.). In that sense, EOs-*β*CD inclusion complexes may be included to develop antimicrobial active cardboard packaging to extend the shelf life of plant products as observed by our Group in vegetables [15,20,24,25]. High relative humidity (RH) and temperatures have been reported to increase the controlled release of EOs from the inclusion complex [20,26], as is expected to occur with the recommended high RH (90%–95%) maintained during cold storage of mandarins and subsequent commercialization during retail at room temperature. Nevertheless, the effects of this antimicrobial active packaging have not been studied yet on fruits with a high potential to increase the shelf life of products usually sold in cardboard packages like mandarins.

This work aimed to study the effect of an active (supplemented with an EOs mix-*β*CDs inclusion complex) cardboard packaging in different formats (different sized trays and boxes) on the mandarin quality after a commercialization simulation (room temperature) at the pilot plant scale up to three weeks (with a previous simulation of short transportation/storage of five days at 8 °C). The selected package was then validated at the industrial scale in a second experiment during storage at 8 °C up to 21 days.

## 2. Materials and Methods

### 2.1. Materials

Carvacrol, oregano, and cinnamon EOs were obtained from Lluch Essence S.L. (Barcelona, Spain). *β*CD (Kleptose^®^10) was obtained from Roquette (Lestrem, France). Waterproof lacquer (UKAPHOB HR 530; 10.5% solids) (authorised for food contact surfaces in accordance with EC (2004) [27]) was acquired from Schill+seilacher GMBH (Böblingen, Germany). Corrugated cardboard was supplied by SAECO company (Molina de Segura, Spain). All microbial analysis materials were acquired from Scharlau Chemie (Barcelona, Spain).

Mandarins (*Citrus reticulata* Murcott Seedless) were obtained from the company Blancasol S.a.t. (Blanca, Murcia, Spain) in March 2019. Mandarins were grown in open fields under organic agriculture practices. Fruits were manually harvested and transported to the pilot plant of our Department, where they were selected according to homogeneous orange colour (more than 80 percent of the surface showed orange colour), physical integrity and absence of decay. No waxes neither fungicides were applied to mandarins. Selected mandarins were then packaged within the corresponding packaging treatment and then stored as described in each experiment (pilot plant scale and industrial validation experiments, as described below).

### 2.2. Preparation of the EOs-βCD Inclusion Complex and Application to Packages

An EOs mix composed of carvacrol:oregano EO:cinnamon EO 70:10:20 (weight (*w*):*w*:*w*) (composition analysis previously reported [25]) was prepared based on its high antimicrobial effect in accordance with our previous studies with several EOs mixes [15,28]. The EOs-*β*CD inclusion complex was prepared using the kneading method [29]. Briefly, 0.15 g of EOs was mixed with 1.14 g of *β*CD (1:1 molar ratio) in a mortar with 3 mL of ethanol, kneaded for 45 min and finally maintained in a vacuum desiccator at room temperature for at least 72 h. The achieved encapsulation efficiency of the EOs-*β*CD inclusion was of 92%–95%, in accordance with our previous data [15]. This EOs-*β*CD inclusion complex has been fully characterized and published by our group [15,25].

The EOs-*β*CD inclusion complex was dissolved in water-diluted lacquer prior to spraying on all internal surface of the package. The lacquer was diluted (to a final solid concentration of 8.5%) to compensate for the addition of EOs-*β*CD inclusion complex since lacquers with solid content >30% make lacquer spraying on the cardboard surface difficult. In that sense, the EOs-*β*CD inclusion complex content was at that maximum concentration that did not compromise the technological properties of the lacquer to be sprayed on the cardboard surface of the packaging in accordance with preliminary tests. Lacquer containing the EOs-*β*CD inclusion complex was sprayed at 12 mL·m^−2^ following the manufacturer’s recommendations to obtain homogeneous spraying on the paperboard surface while reaching a maximum lacquer absorption. The mechanical and hydrophobic properties of the paperboard material, sprayed and non-sprayed, with the EOs-*β*CD inclusion complex are fully described in our previous publication [15].

### 2.3. Design of the Pilot Plant Experiment and Industrial Validation Experiment: Packaging Treatments and Storage Conditions

Two experiments were performed during this study: pilot plant scale experiment and industrial validation experiment.

#### 2.3.1. Experiment 1: Pilot Plant Scale

**Figure 1:**
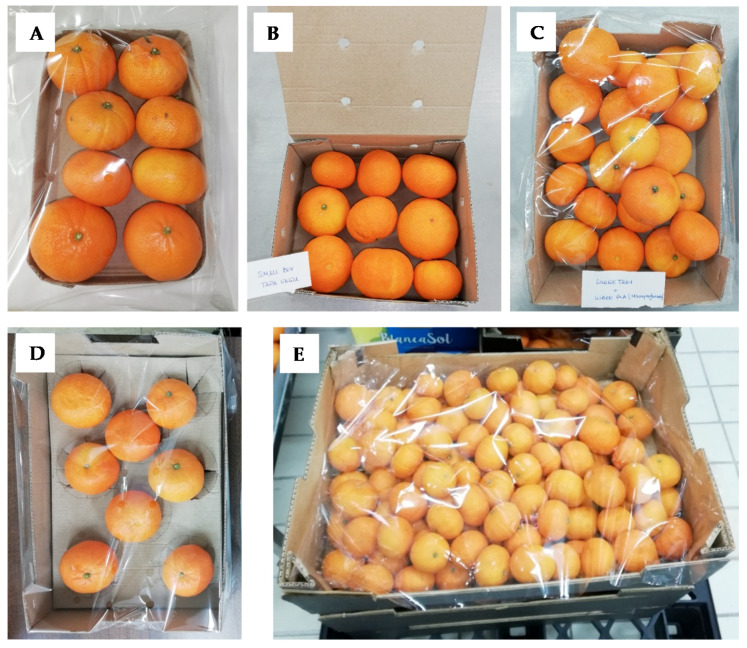
Package formats studied: (**A**) small tray (ST); (**B**) small box (SB); (**C**) large tray (LT); (**D**) large tray+alveoli (LT+); E, large box (LB).

The first experiment was developed at pilot plant scale studying five different packaging formats to select the most appropriate in order to highly maintain the mandarin quality during a short cold storage period (simulating a short transport/storage of 5 days at 8 °C) supplemented with a commercialization period at room temperature up to 3 weeks. The five package formats (Figure 1) were:

- Small tray (ST): Tray (230 × 120 × 25 mm; length × width × height) of microcorrugated paperboard with flow-pack of macroperforated (45 holes (8 mm Ø each) per m^2^) film of polylactic acid (PLA). Each ST contained 0.7–0.8 kg of fruit.
- Small box (SB): Box with lid (190 × 150 × 75 mm) of macrocorrugated paperboard. Each lateral side of the box had 2 holes (10 mm Ø each) and the lid 4 holes (10 mm Ø each). Each SB contained 0.7–0.8 kg of fruit.
- Large tray (LT): Tray (300 × 200 × 90 mm) of macrocorrugated paperboard with a cover of the macroperforated PLA film. Each LT contained 2.3–2.4 kg of fruit.
- Large tray+alveoli (LT+): LT with alveoli (also known as pulp tray) of microcorrugated paperboard. Each LT+ contained 8 mandarins.
- Large box (LB): Box (600 × 400 × 180 mm) of macrocorrugated paperboard with a cover of the macroperforated PLA film. Each LB contained ≈10 kg of fruit.

Each of the five package types was also tested as active or non-active packages. For active packages, all internal surfaces of paperboard packages were sprayed with the lacquer containing the EOs-*β*CD inclusion complex (as described in the previous section). For the active LT+, only the alveoli tray was sprayed with the lacquer containing the EOs-*β*CD inclusion complex. For non-active packages, the same procedure was conducted but the lacquer did not include the EOs-*β*CD inclusion complex.

All packages with fruit were stored in this first experiment during a cold storage (8 °C, 90%–95% RH) for 5 days (simulating a short transport/storage) followed by a simulated commercialization period (at room temperature and ambient RH) up to 3 weeks. Three replicates (three packages) per each of 10 packaging treatments (5 packaging types×package activity (non-active and active)) were prepared per each of the following 4 sampling times: 5 days-cold storage (CS), CS+1 week (wk) of commercialization (CS+1 wk), CS+2 wk and CS+3 wk. Then, a total of 120 packages were prepared. All analyses and determinations were conducted in our laboratory of the Universidad Politécnica de Cartagena.

#### 2.3.2. Experiment 2: Industrial Validation

Once the effect of a short cold storage+commercialization period was studied in Experiment 1, the Experiment 2 studied the effect of the selected package type (LB, as described in “Results” section) during a long cold (8 °C) storage period up to 21 days (which may also simulate a transatlantic transport). For it, harvested mandarins were packaged in the LB (≈10 kg of fruit per cardboard box) (active or non-active) in the company (Fruca S.A.; Beniaján, Murcia, Spain) installations by the company personnel as usually conducted in pallets (83 cardboard boxes per pallet). Then, the packaged product was stored in the company cold rooms at 8 °C and 90%–95% RH.

Three replicates (three packages) per each 2 packaging treatments (non-active LB and active LB) were taken per each 3 sampling times: 7, 14, and 21 days. At each sampling time, three LB packages with fruit were taken from the company installations and transported to our laboratory of the Universidad Politécnica de Cartagena where all analyses and determinations (except weight loss, which was always monitored in the company cold rooms) were conducted.

### 2.4. Weight Loss

Weight of packages containing the product was monitored at each sampling time to determine the weight loss (%) of mandarins during storage.

### 2.5. Soluble Solids Content and Titratable Acidity

Juice from mandarin wedges was obtained with a blender (model MX2050; Braun, Germany). SSC was determined with a digital handheld refractometer (Atago N1; Tokyo, Kanto, Japan) at 20 °C and expressed as °Brix. TA of the diluted juice (2 mL plus 48 mL of distilled water) was determined with an automatic titrator (model T50; Metter Toledo; Milan, Italy) with 0.1 M NaOH to reach pH 8.1. *TA* was expressed as citric acid in %. *BrimA* index (Equation (1)) [5] was selected as the most appropriate maturity index for mandarins as recommended for citrus [6].

(1) BrimA=SSC−(k×TA)

where *k* is the tongue’s sensitivity index, normally ranging from 2 to 10 [5], set at value 3 for citrus [6]. Each of the three replicates was analysed in duplicate.

### 2.6. Colour

Colour of mandarin (external colour) was determined using a colourimeter (Chroma Meter CR-400, Konica Minolta, Japan) at illuminant D65 and 2° observer, and with a viewing aperture of 8 mm. Three measurements were made (equatorial zone) per each fruit and they were then automatically averaged by the device. Ten mandarins were analysed per each replicate. The following specific colour index (Equation (2)) was selected as the most appropriate for orange citrus as previously reported [11,30]:

(2) $$Colour\;index=\frac{1000\times a^{*}}{L^{*}\times b^{*}}$$

where L*, a*, and b* refer to the CIE colour parameters obtained from the colourimeter measurements.

### 2.7. Firmness

Firmness was determined with a texture analyzer (model TA XT Plus; TA Instruments; Surrey, UK) by measuring the amount of force (N) to compress 8-mm deep (P/10 probe of 10 mm Ø) a whole mandarin fruit on the diameter height. The texture analyzer was set for a drop speed of 20 mm min^−1^ and equipped with a load cell of 5 kg. Ten mandarins were analysed per each replicate.

### 2.8. Microbial Analyses and Decay Incidence

Surface microbial loads of mandarins were analysed as previously described [24]. Briefly, three mandarin fruits were mixed with buffered peptone water (1:1 *w*:volume) and then homogenised for 1 h at 120 rpm in an orbital shaker at 4 °C. Viable counts were based on duplicate counts by 10-fold serial dilutions in buffered peptone water. Then, aliquots (1 mL) of the microbial dilutions were pour-plated in plate count agar and violet red bile dextrose agar for mesophiles and psychrophiles and enterobacteria, respectively. For yeast and moulds, microbial aliquots (0.1 mL) were spread-plated on rose bengale agar. Mesophiles, psychrophiles, enterobacteria, yeast, and moulds were incubated at 31 °C (48 h), 4 °C (7 days), 37 °C (24 h), 25 °C (5 days) and 25 °C (7 days), respectively. Results were expressed as log colony forming units (CFU) cm^−2^. Each of the three replicates was analysed in duplicate.

Decay incidence was quantified by calculating the percentage of rotten mandarins (fruits with visible mycelial growth) independently in each of replicates (packages). 

### 2.9. Sensory Analyses

Sensory analyses were performed according to international standards [31]. Sensory tests were conducted in a standard room [32] equipped with ten individual taste booths. The panel consisted of twelve assessors (six women and six men, aged 22–61 years) who had been trained in discriminative quality attributes. Mandarin segments (5 units) were served at room temperature in transparent glass plates coded with three random digit numbers. Still mineral water was used as a palate cleanser. The quality attributes scored were overall quality (5: excellent; 3: fair, limit of acceptability (below 3 were not sensory accepted); 1: extremely bad), overall flavour (5: excellent; 3: fair; 1: extremely bad), sweetness (5: very sweet; 3: fair; 1: little sweet), acidity (5: very acid; 3: fair; 1: little acid) and juiciness (5: very juicy; 3: fair; 1: little juicy). The product shelf life was established based on the limit of acceptability of the product overall quality.

### 2.10. Statistical Analyses

The data were subjected to analysis of variance (ANOVA) using the SPSS software (v.19 IBM, New York, NY, USA). Statistical significance was assessed at *p* = 0.05, and the Tukey’s multiple range test was used to separate the means.

## 3. Results

### 3.1. Experiment 1: Selection of Active Package (Pilot Plant Scale)

#### 3.1.1. Weight Loss

The weight loss of samples after the cold storage period (CS; 5 days at 8 °C) was very low (<1.5%) (Table 1). No significant (*p* > 0.05) differences (SSC, TA, neither BrimA) among non-active and active samples were observed for any of the packaging treatments after the CS period (Table 1). Similarly, low weight losses have been reported in mandarins after short cold storage periods (<1 week) showing the adopted low storage temperature in cold rooms, together with high RH, a crucial role to slow down the physiological processes responsible for product weight losses (mainly due to dehydration) [33,34,35]. Nevertheless, mandarins are stored at room temperature for long periods during retail, leading to increased weight loss [36].

Weight loss of mandarins was increased during the commercialization period having all factors (packaging type, package activity and storage time), and their interactions, statistically significant effect on weight loss (*p* < 0.001) (Table 1). In accordance, mean (packaging type-package activity) weight losses of 3.3, 8.1, and 11.6 after CS+1 wk, CS+2wk, and CS+3wk, respectively, were observed. In particular, samples within non-active LB and LT showed the highest weight losses (14–16%) after CS+3wk, which may be attributed to the high product weight:package surface rates of these packages. Weight losses were significantly (*p* < 0.001) reduced using active packages, compared to non-active packages, with weight losses of 8%–9.5% after CS+3wk RT. Nevertheless, samples within active SB registered the highest weight loss (12.9%) among active samples after CS+3wk RT, probably due to the numerous and large perforations of this package that possibly intensified the weight loss in these mandarins. On the other side, the use of active LB and LT led to the highest weight loss reductions (decreases of 5.2 and 6.3 weight loss units, respectively) compared to their respective non-active samples after CS+3wk RT. In that sense, the controlled EOs release from active packages highly reduced the product weight loss for LB and LT. That finding denotes the benefits of the use of a macroperforated liner (LB and LT) compared to the macroperforated cardboard cover (SB), apart from the reduced visibility of the product for the consumer.

The transpirational water loss from the primary surface of plant products is limited by the plant cuticle as observed in citrus [37]. The chemical composition and/or the spatial arrangement of the components of the cuticle are more linked to other physical parameters like cuticle thickness or wax coverage [38]. Hence, the released EOs might change such cuticle properties, as previously studied [39], probably leading to the hereby observed lower weight loss of mandarins using the active packages.

#### 3.1.2. Soluble Solids, and Titratable Acidity

Mandarins showed initial SSC and TA of 15.1 °Brix and 1.23%, respectively, at day 0 (Table 1). The individual factors package type and storage time were significant for SSC and TA, while package activity factor did not (Table 1). All factor interactions were also significant for SSC and TA (except packaging type×package activity for TA). SSC was decreased after the CS period by 1.6–2 °Brix showing LT and SB samples the highest SSC reductions with mean values (averaged between non-active and active) of 2.4 and 2 °Brix, respectively. In contrast, TA values of samples increased by 0.3–0.4 TA units after the CS period, without great differences (<0.2 TA units) between packaging treatments.

The cold storage of horticultural products is interpreted by plant cells as an abiotic stress leading to the synthesis of antioxidant compounds (e.g., organic acids), as previously reported in citrus fruit [40], during the first days of cold storage. Lately, the biosynthesis rates of these antioxidants decrease. In that sense, the observed TA increment and SSC reduction during cold storage may be explained by the organic acids biosynthesis using sugars as energy pools [15].

During the commercialization period, SSC increased, while TA was reduced, showing mandarins differences of 0.11–0.38 TA units after three weeks. Particularly, active LB samples showed the highest TA reduction of 0.38 TA units. Mature citrus fruits, which are classified as non-climacteric, evolve very low amounts of ethylene during ripening and a low respiration rate, but respond to exogenous ethylene by ripening-related pigment changes and accelerated respiration [41,42]. Furthermore, the ethylene biosynthesis pattern described as system I, typical from non-climateric fruit, revealed that the use of 1-Methylcyclopropene (a chemical competitive inhibitor of the ethylene active sites of plant products) in citrus resulted in an increase in ethylene evolution [41]. It has been demonstrated that EOs inhibited ethylene biosynthesis in several fruit and vegetables, although such mechanism is not still fully understood [43,44,45,46,47,48]. In contrast, it has been already demonstrated a competitive inhibition of EOs within the active sites of browning-relevant enzymes (polyphenol oxidase, peroxidase and phenylalanine ammonia-lyase) in lettuce [49]. In that sense, the inhibitory effect of EOs on ethylene production could be owed to a competitive inhibition of the EOs with the active sites of key enzymes of the ethylene biosynthesis pathway (aminocyclopropane-1-carboxylic acid synthase and Met-adenosyltransferase). Overall, EOs action could produce the same effect as 1-Methylcyclopropene in citrus [41] leading to an acceleration of senescence and respiration with the consequent consumption of organic acids and sugars increment (leading to an incremented maturity index as shown below) as reported in citrus fruits like mandarins [50,51]. TA differences >0.36 TA units have been reported to be significantly detected in sensory analyses [51]. Furthermore, reduced TA and increased SSC occurred during fruit postharvest life led to higher sensory scores and greater consumer acceptability of mandarins and oranges [6,51,52].

Due to the high importance of a balanced SSC-TA binomial for the fruit quality, the SSC/TA maturity index has been conventionally adopted. Nevertheless, BrimA index has been proposed as a better predictor of flavour for citrus fruits since SSC/TA is calculated as a ratio, rather than a subtractive calculation like BrimA [5,6]. The *k* value (Equation (1)) reflects the tongue’s higher sensitivity to acid than to sugar allowing this index that smaller amounts of acid than sugar to make the same numerical change to BrimA, and in opposite direction. The BrimA index works on the simple principle that sugar and acid tend to have opposing effects on taste and the tongue perceives sugar and acids with differing sensitivities [5,53]. BrimA reached values of 11–13 at the third week of commercialization (Table 1). Particularly, active LB, LT and SB showed BrimA values below 11.5, showing active LB the lowest value (10.9), while the rest of the samples showed BrimA values above 11.5. Such trends are in accordance with sensory scores of active LB, LT, and SB after CS+3 wk period (see “Sensory analyses” section).

In conclusion, LB samples showed the highest TA reduction, which may be sensorially positive-appreciated, during commercialization while LT and SB samples showed the highest SSC reductions during the cold storage period, which may reduce their sensory acceptance.

#### 3.1.3. Firmness

Mandarins showed an initial firmness of 19.3 N (Table 1). The individual factor storage time was significant (*p* < 0.001) for the fruit firmness. In that sense, a general decrease of ≈3 N was observed after the second week of commercialization when comparing mean values (average of all sample treatments) with day 0. The firmness reduction of mandarins, and other citrus fruits, during postharvest storage has been well correlated to the cellular wall polysaccharides, which may be decreased during fruit maturation [12]. The individual factor packaging type was also significant (*p* < 0.01) for firmness. Particularly, mean firmness values (averaged between active and non-active during all storage time) of LB samples showed the highest firmness (18.4 N), while SB showed the lowest mean firmness value (17.3 N). The benefit from using alveoli trays (LT+) on the fruit firmness was not hereby observed since such differences are only appreciated after vibrations occurred during a real transport. On the other side, the individual factor package activity was not significant (*p* > 0.05) for fruit firmness, neither the double interaction package type×package activity nor the triple interaction.

Overall, LB samples showed the highest firmness during storage, regardless of active or non-active packages. Nevertheless, our previous studies with tomatoes showed that product firmness was better maintained during storage when using active packages including the same EOs-*β*CD inclusion complex [24,25]. The latter finding may be explained since the tomato peel is more susceptible to dehydration processes during postharvest life than mandarin peel, which has the characteristic oil vesicles of citrus fruits that may provide higher mechanical resistance to the fruit.

#### 3.1.4. Colour

Mandarins showed initial CIE colour parameters of L* = 59.5 ± 1.0, a* = 59.5 ± 1.0, b* = 59.5 ± 1.0, which corresponded to Chroma = 67.6 ± 2.7 and °Hue = 65.0 ± 1.6 (Supplementary Material Table S1). A marked luminosity (L*) increase and a mild yellowness increment (b*) were observed in the first week of commercialization after the cold storage period, which was correlated with a CI decrease after CS+1 wk (Table 1). Such colour changes are due to the accumulation of carotenoids (up to 94% present in the mandarin peel) after colour beak of the fruit, specially β-cryptoxanthin and apocarotenoids, which are responsible for the distinctive orange-reddish pigmentation in mandarins [54].

CI is a good colour index for citrus fruits that reflect all L*, a* and b* changes together as previously reported [30]. It showed similar trends to a*/b* index, another classical colour index in citrus fruits [7]. The individual factor package type was significant (*p* < 0.05) for CI showing LT the highest colour changes after the first week of commercialization, while LB and LT+ showed the lowest CI changes. The observed CI differences among LT and LT+ may be explained since lower quantity of fruits, and more spaced, were disposed within the LT+ package probably leading to a lower ethylene accumulation, the ripening hormone with autocatalytic nature [55].

Later, as the commercialization period advanced, CI was increased to levels comparable to day 0 and CS. It may be explained by the mild redness (a*) increment observed in such advanced commercialization stage, which agrees with the orange to red-orange turning during mandarin maturation, highly contributing a* in the CI equation (Equation (2)). In contrast, package activity factor was not significant (*p* > 0.05) for colour parameters (L*, a*, b* nor CI), neither the double, nor triple, interactions with this factor.

As observed, no great colour changes were observed during cold storage of mandarins, showing LB and LT+ the lowest colour changes after the first week of commercialization. Furthermore, the active material did not negatively affect the fruit colour during storage.

#### 3.1.5. Sensory Analyses

Sensory quality of mandarins, and particularly flavour, is highly influenced by attributes like sweetness and acidity. Furthermore, the flavour acceptance of mandarins is the result of an appropriate high sweetness combined with a pleasant low-moderate acidity. In that sense, sensory analyses of this study were focussed on the determination of this complex attribute that is the fruit flavour. Meanwhile, other attributes like colour and consistency are more accurately determined by colour and firmness measurements, as previously showed.

**Figure 2:**
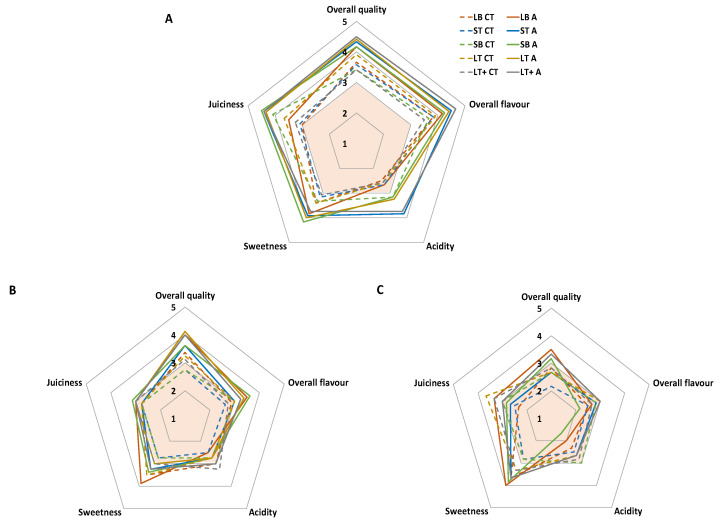
Sensory scores of mandarins packaged within different packages (ST, small tray; SB, small box; LT, large tray; LT+, large tray with alveoli tray; and LB, large box), non-active (CT) and active (A), after cold storage ((**A**); 5 days at 8 °C) followed by a commercialization simulation (room temperature) for 2 weeks (**B**) or 3 weeks (**C**). The coloured orange area defines the limit of acceptability (scores below 3 are considered below the limit of acceptability).

After the CS period (Figure 2), all samples showed overall quality scores over the limit of acceptability, reaching the highest scores mandarins within active packages (4.2–4.5), while for non-active ones were closer to the limit of acceptability (3.4–3.9). Particularly, samples within active packages showed the highest sweetness scores (3.8–4.2) after CS period, without great differences among packaging treatments, showing active LB the lowest acidity score (2.7). The tongue reflects a higher sensitivity to acid than to sugar [53]. Furthermore, reduced acidity, together with increased sweetness, have been positively scored by panellists during maturation of oranges [6,51]. In that sense, the lower acidity in such active samples probably explains the higher sweetness appreciation of these samples after the CS period. Such reduced acidity of LB samples may be explained by the higher product weight:package surface rate of this package, which may lead to a higher autocatalytic synthesis of ethylene, with the consequent ripening effects, like the reduction of organic acids due to the product respiration that has been reported to occur in citrus fruit [50,51]. Nevertheless, ripening must be also controlled showing the active packaging a crucial role due to the reported properties of EOs to control ethylene biosynthesis in fruits [43]. No off-flavours, nor those related to EOs (due to the controlled and low EOs release from these active packages [20,24]), were detected during both CS and commercialization periods.

As expected, fruit maturation was enhanced during the commercialization period, characterized by a more pronounced acidity reduction coupled with a sweetness perception increment. Active samples showed higher sweetness and lower acidity compared to non-active samples. All non-active samples registered overall quality scores below the limit of acceptability after CS+3wk. Attending to active samples, those with higher product weight:package surface ratio (LB, LT and SB) reached overall quality scores over the limit of acceptability, displaying active LB the highest overall scores (3.5).

In conclusion, the shelf life of mandarins during a commercialization period (following a previous cold storage of five days at 8 °C) could be established in three weeks for active packages with high product weight:package surface ratio. Particularly, active LB reached the highest sensory scores, while it is reduced to two weeks for non-active packages. 

#### 3.1.6. Microbiology and Decay Incidence

Mandarins showed initial mesophilic, psychrophilic, enterobacteria, yeasts and moulds loads of 1.3, 1, <0.5, 2.3 and 2.2 log CFU cm^−2^, respectively (Table 2). In general, microbial loads increased during storage periods, although such microbial growth was low. In that sense, microbial loads of samples were below 2.8 (mesophilic), 2.6 (psychrophilic), 1.6 (enterobacteria), 2.4 (yeasts) and 3.5 log CFU cm^−2^ (moulds) after CS+3wk. Such low surface microbial loads are usual in fresh fruits, compared to processed (e.g., fresh-cut) fruits. The higher microbial loads corresponded to moulds. In particular, *Alternaria* and *Cladosporium* have been reported as the main moulds in mandarins, followed by *Fusarium* and *Rhizopus* [56].

All individual factors (package type, package activity and storage time) were significant (*p* < 0.001) for all microbial groups. In general, samples within active packages showed lower microbial loads than their respective non-active packaged samples. Furthermore, double interactions with storage time were also significant (*p* < 0.001) for all microbial groups.

Mesophilic loads augmented by 0.5–1 log units after the CS period, showing active LB the lowest load with 1.2 log CFU cm^−2^. Mesophilic loads of non-active packaged samples incremented by 1–1.1, 1.2–1.4 and 1.4–1.6 log units after one, two, and three weeks of commercialization, respectively, for LB and ST packages. Meanwhile, the rest of non-active packaged mandarins registered increments of 1–1.4 during the three-week commercialization without high differences among them. Although non-active LB and non-active ST samples showed the highest mesophilic increments, their respective active packages highly reduced the mesophilic increments after CS+3 wk by two and 2.7 fold, respectively. In that sense, samples packaged within active packages showed similar (*p* > 0.05) mesophilic loads of 1.7–1.9 log CFU cm^−2^ after CS+3 wk.

In general, no significant (*p* > 0.05) psychrophilic increments were observed after the CS period, except non-active LT and non-active LB that registered psychrophilic growth of 1.4–1.5 log units. The higher growth in these packaging treatments may be explained by the high quantity of samples per package, which may stimulate the microbial growth due to the high humidity and low ventilation, especially in the fruits in the bottom of the package. Nevertheless, the active LT and active LB packages showed 1–1.7 lower log units after the CS period, compared to their corresponding non-active packages. No significant (*p* > 0.05) psychrophilic growth was observed during the first 1 week of commercialization. At the third week of commercialization, psychrophilic increments of 1–1.5 were observed for non-active ST, SB and LT, compared to their respective initial loads, although their respective active versions did not register significant changes (*p* > 0.05). Attending to their final loads, active LB and active LT+ registered the lowest psychrophilic loads of 1–1.3 log CFU cm^−2^ after CS-3wk.

Enterobacteria loads were below 1 log CFU cm^−2^ after the CS period, except non-active SB and LT with loads of 1.2 and 1.6 log units, respectively. After the first week of commercialization, enterobacteria loads of non-active packages increased by 1–1.7 log units, while no significant (p > 0.05) changes were observed for active packages. Enterobacteria loads did not change (p > 0.05) during the second and third week of commercialization, compared to the first week, being kept the latter trend related to the effectiveness of active packages. In that sense, active packages showed enterobacteria loads below 1 log CFU cm^−2^ after 3 weeks of commercialization. 

Mould loads of samples did not register high changes (<0.8 log units) after CS, CS+1 wk and CS+2 wk periods. Non-active LB, ST, and LT showed mould increments of 1–1.3 log CFU cm^−2^ after CS+3 wk, compared to their respective initial levels. In general, active samples showed 0.5–1 fewer log units than their respective non-active samples at CS+3 wk. Attending to yeasts, low changes (<1 log units) were observed during both cold storage and three weeks of commercialization. As observed, low yeast and moulds changes were observed during storage periods of mandarins due to the lower growth rate of these microbial groups.

Attending to decay incidence, the storage time factor was not significant (*p* > 0.05), neither their double and triple interactions with package type and package activity (data not shown). Mean values of samples during the complete commercialization period did not show significant (*p* > 0.05) differences among them, with decay incidence values ranging between 1.3% and 7%. The absence of the expected increment of decay incidence throughout commercialization may be due to the low quantity of fruits per packages, especially for ST, SB and LT+. For the same reason, no conclusions can be obtained related to the effect of package type and package activity factors on the decay incidence of samples.

As observed, no microbial growth (except mesophilic loads that slightly increased by 0.5–1 units) was observed after the 5-days cold storage at 8 °C, which remarks the importance of cold storage to extend the shelf life of citrus fruit attending to microbial quality [13]. Nevertheless, fruits are usually commercialized during retail at temperatures higher than 8 °C, usually at room temperature for the case of citrus fruits. Particularly, microbial growth of 1–1.6 log units was achieved after 3 weeks of commercialization simulation (with the previous cold storage) reaching mesophiles the highest increments at this room temperature period. Nevertheless, active packages highly controlled the microbial growth, especially high in non-active LB, but active LB better controlled the microbial growth among the rest of active packages. In that sense, the controlled EO release from the *β*CD inclusion complex (as characterized in our previous publications [20,24]) led to an antimicrobial effect, which was more appreciable during the commercialization period since at such higher temperatures the EO release from the inclusion complex is higher. The latter behaviour is explained since molecular Brownian motion is enhanced with the temperature increments, leading to a higher EO release from the inclusion complex [57].

As far as microbial growth is concerned, enterobacteria seemed to be more sensitive to the released EOs from active packages, as previously observed in tomatoes by our Group [20,28]. Carvacrol, the major component of the hereby used EOs mix of the complex, was considered, together with thymol (the major component of oregano EO together with carvacrol), as the EOs component with the widest spectra activity. In that sense, carvacrol is effective against both gram-negative and gram-positive with even higher effectiveness against the gram-negative bacteria (e.g., enterobacteria) [17,58]. In that sense, the hydroxyl group from the structure of this phenolic compound is crucial to disintegrate the outer membrane of gram-negative bacteria, which is more susceptible to the EOs antimicrobial properties [18]. Furthermore, the higher susceptibility of gram-negative bacteria to EOs has been hypothesized due to the less dense cell wall and lower peptidoglycan content compared to Gram-positive bacteria, as observed in a study with oregano and thyme EOs [58].

### 3.2. Experiment 2: Industrial Validation of the Selected Active Packaging

In accordance with experiment 1, LB was selected for the industrial validation experiment owed to the high beneficial effects of active LB on the mandarin quality to reach a long shelf life. Once the quality changes were studied during the most adverse storage conditions for mandarins (i.e., room temperature storage), the industrial validation experiment was studied at a recommended cold storage for mandarins [13]. 

#### 3.2.1. Weight Loss

**Figure 3:**
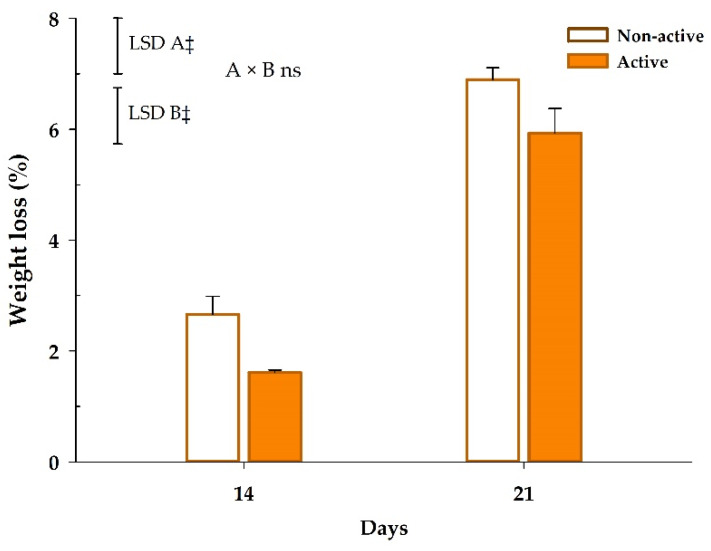
Weight loss of mandarins packaged within non-active and active packages up to 21 days at 8 °C (*n* = 3, ± SD). Least significant differences (LSD) are represented with bars. The uppercase letters A and B denote package type and storage time, respectively. ns, not significant (*p* > 0.05).‡ significance for *p* ≤ 0.001.

The weight loss of samples after 7 days was very low (<1%), as previously observed in the experiment 1. Package activity and storage time individual factors were significant (*p* < 0.001) for the weight loss of samples (Figure 3). In that sense, samples within active packages showed 1–1.1 fewer weight loss units than non-active packaged mandarins during storage. As expected, weight losses increased during storage with an increment of 4.2–4.3 weight loss units after 14 and 21 days, respectively. Nevertheless, no significant differences (*p* > 0.05) were observed between non-active and active samples.

Overall, weight losses of samples within non-active and active packages during storage were low after 14 days (2.7% and 1.6%, respectively). Weight losses were then incremented to 6.9% and 5.9% for non-active and active samples, respectively, after 21 days at 8 °C. As previously discussed, the released EOs may cause mild structural changes in the structures of the fruit surface [39], such as in the cuticle, leading to a lower water mass transport in accordance with weight loss data.

#### 3.2.2. Soluble Solids Content, Titratable Acidity and Firmness

**Figure 4:**
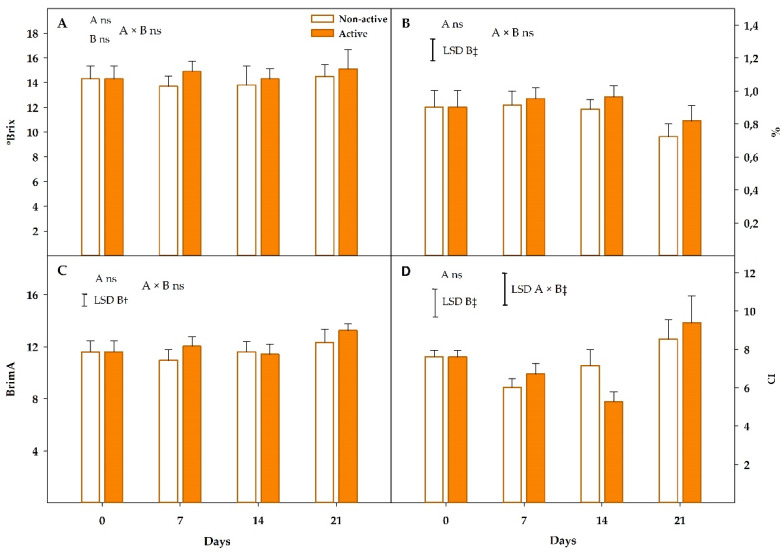
Soluble solids content (**A**), titratable acidity (**B**), BrimA maturity index (**C**) and colour index (**D**) of mandarins packaged within non-active and active packages up to 21 days at 8 °C (n = 3 ± SD). Least significant differences (LSD) are represented with bars. The uppercase letters A and B denote package type and storage time, respectively. ns, not significant (*p* > 0.05).† and ‡ significance for *p* ≤ 0.01 and 0.001, respectively.

Mandarins showed initial SSC, TA, and BrimA values of 14.3 °Brix, 0.9%, and 11.6, respectively, at day 0 (Figure 4A–C). Package activity and storage time effects, as well as their interaction, were not significant (*p* > 0.05) for SSC, with SSC values of 13.7–15.1 °Brix during all cold storage period (Figure 4A). The observed slight SSC reduction during the five day-cold storage period (experiment 1) was not hereby observed, since this antioxidant response, which implies the use of sugars as an energy source [15], was probably downregulated from day 5 to day 7.

As far as TA is concerned, the package activity, and its interaction with storage time, did not show significant (*p* > 0.05) effects on TA (Figure 4B). In general, no significant changes (*p* > 0.05) were observed between active and non-active samples although non-active samples showed a TA value 0.18 units lower after 21 days of storage. Samples packaged within the active package did not show (*p* > 0.05) such TA decrease, but a similar trend was observed. This TA decrease is common during citrus fruit maturation, as previously discussed due to the respiratory activity of the plant product [50,51], and it was positively correlated with a higher sensory acceptability of mandarins [6,51,52]. Such respiratory rates are higher at room temperature compared to the cold storage temperature, since a significant TA reduction was only observed after 21 days at 8 °C.

As expected, the ripening of mandarins advanced during cold storage in accordance with BrimA values (*p* < 0.01), although in a lower rate compared to the commercialization period (experiment 1). In that sense, significant BrimA changes were only observed after 21 days of storage at 8 °C (Figure 4C). Contrary to storage time, the package activity factor did not show a significant effect (*p* > 0.05) on BrimA maturity index, neither the package activity×storage time interaction.

Mandarins showed firmness values of 16.2–18.7 N during cold storage, with no significance (*p* > 0.05) for any of the individual factors and their interaction (data not shown).

Conclusively, the use of the active package did not affect the SSC and TA attributes during storage at 8 °C, which may not compromise the consumer acceptance due to the high importance of these attributes on the flavour acceptance of mandarin samples [6]. Furthermore, the firmness of mandarins was not affected during cold storage, independently of the packaging treatment.

#### 3.2.3. Colour

Mandarins showed initial CIE colour parameters of L* = 64.1 ± 0.7, a* = 30.0 ± 1.3, b* = 61.6 ± 1.3 in this experiment 2, which corresponded to Chroma = 68.5 ± 1.5 and °Hue = 64.0 ± 0.9 (Supplementary material Table S2). The storage time factor was significant (*p* < 0.001) for all L*, a* and b*. These colour parameters were reduced during storage. Particularly, L* and a* showed low and moderate reductions of <3 and <9 units after 21 days of cold storage, respectively, while b* was reduced by 6 and ≈20 units after 7–14 and 21 days, respectively. These colour changes corresponded to an increment of CI during cold storage, reaching CI increases in active samples of 1.8–2 units after 14–21 days (Figure 4D), while such CI increments were not significant (*p* > 0.05) for non-active samples. As observed, active packages still allowed a moderate fruit turning from orange to red-orange, as a result of the carotenoids biosynthesis [54]. Nevertheless, these colour changes were not high, which would not compromise the consumer acceptance (see “Sensory analyses” section).

#### 3.2.4. Sensory Analyses

**Figure 5:**
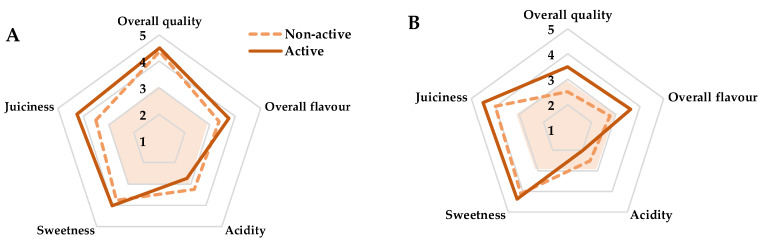
Sensory scores of mandarins packaged within active or non-active package after 14 days (**A**) and 21 days (**B**) of storage at 8 °C. The coloured orange area defines the limit of acceptability (scores below 3 are considered below the limit of acceptability).

Sensory analyses of samples after 14 days of storage showed good sensory scores without high differences (<0.5) between samples stored within non-active and active packages (Figure 5). Particularly, mean values (non-active and active) for overall quality, overall flavour, acidity, sweetness and juiciness of 4.4, 3.6, 3, 4, and 3.9, respectively, were observed after 14 days.

After 21 days, samples within active packages still showed sensory scores over the limit of acceptability, with overall quality and overall flavour scores of 3.5 and 3.6, respectively, while non-active samples were not sensorially accepted (Figure 5). Furthermore, active samples showed lower acidity scores than non-active samples, which is in accord with TA data, which might lead to a higher consumer acceptance [6,51].

In conclusion, the shelf life of mandarins during storage at 8 °C could be established, based on sensory quality related to flavour attributes, in at least 21 days for samples stored within the active package, while such shelf life is reduced to 14 days when mandarins are stored within non-active packages.

#### 3.2.5. Microbiology and Decay Incidence

**Figure 6:**
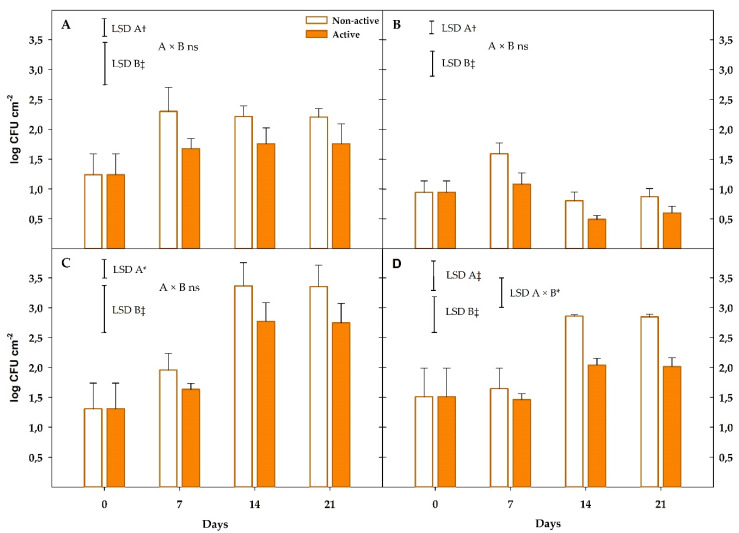
Microbial loads (**A**), mesophiles; (**B**), enterobacteria; (**C**), moulds; (**D**), yeasts of mandarins packaged within non-active and active package up to 21 days at 8 °C (*n* = 3, ± SD). Least significant differences (LSD) are represented with bars. The uppercase letters A and B denote package type and storage time, respectively. ns, not significant (*p* > 0.05). *, † and ‡ significance for *p* ≤ 0.05, 0.01 and 0.001, respectively.

Mandarins also showed low initial loads (1–1.5 log CFU m^−2^) for all microbial groups (Figure 6), as previously explained in Experiment 1. The storage time factor was significant (*p* < 0.001) for all microbial groups, except for psychrophiles.

Attending to mesophiles, an increment of 1 log unit was observed after seven days in samples stored within the non-active package, whose loads were maintained (*p* > 0.05) during the rest of storage period (Figure 6A). Package activity factor was significant (*p* < 0.01) for mesophiles, with loads ≈1.7 log units lower at every sampling time compared to the non-active samples.

Psychrophiles loads of 0.5–1.3 log CFU m^−2^ were observed during all storage periods without significant (*p* > 0.05) changes among the different sampling times (data not shown). Furthermore, package activity factor was not significant (*p* > 0.05) for psychrophiles loads.

Enterobacteria growth was also very low during storage with the only significant increment observed at day 7 (Figure 6B). Package activity factor was significant (*p* < 0.01) for enterobacteria loads, showing samples within active packages 0.5–1.1 lower log units than non-active samples. As observed, enterobacteria growth at 8 °C was low due to the higher optimum growth temperatures for this microbial group. Although such lower enterobacteria growth at this storage temperature did not allow to show the higher effectivity of EOs against this microbial group, as observed in Experiment 1, mandarins within active packages showed the lowest enterobacteria loads (0.6 log CFU m^−2^) after 21 days at 8 °C.

Moulds showed the highest growth among microbial groups with 2 and 1.5 higher log units after 21 days for non-active and active samples, respectively (Figure 6C). Particularly, the highest growth was observed from day 7 to day 21, while no significant growth (*p* > 0.05) was detected in the first seven days of storage. As observed, the package activity factor was significant for mould loads, showing mandarins within the active package ≈0.6 lower log units than non-active samples at days 14 and 21. The lower optimum growth temperature for moulds allowed the observed higher growth, compared to other groups like enterobacteria, although active packages still controlled such mould growth.

Similar to moulds, package activity and storage time factors were significant (*p* < 0.001) for yeasts (Figure 6D). In that sense, yeast growth of 1.4 log units was observed in non-active samples, while increments of only 0.5 units were observed in active samples after 21 days.

**Figure 7:**
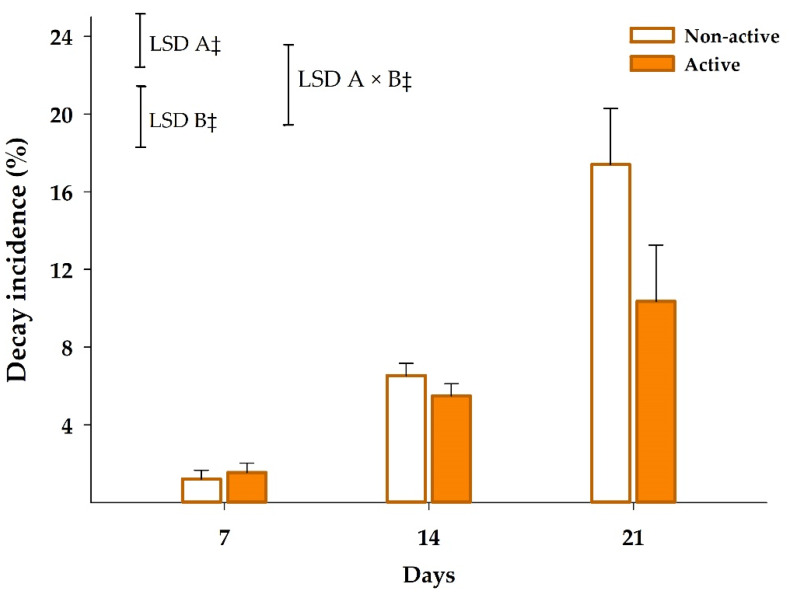
Decay incidence of mandarins packaged within non-active and active package up to 21 days at 8 °C (*n* = 3, ± SD). Least significant differences (LSD) are represented with bars. The uppercase letters A and B denote package type and storage time, respectively. ‡ significance for *p* ≤ 0.001.

Decay incidences of 1.1%–1.6%, 5.5%–6.5% and 10.4%–17.4% were observed after seven, 14 and 21 days, respectively (Figure 7). Package activity and storage time factors, as well as their interaction, were significant for decay incidence (*p* < 0.001). Mandarins within active packages showed lower decay incidence that non-active samples. Furthermore, this benefit was even enhanced as the storage time increased with a two-fold reduction of decay incidence after 21 days.

In conclusion, the controlled release of EOs from the βCD inclusion complex of active boxes allowed to control microbial growth of mandarins packaged within this active package, which was also reflected on the reduced decay incidence. Furthermore, the inhibition of ethylene production by EOs [43,44] may also be responsible for the observed decay incidence reduction since ethylene may promote fungus growth as widely reported [59].

## 4. Conclusions

The use of active packaging including *β*CD-EOs inclusion complex extended the shelf life of mandarins during a commercialization simulation (room temperature; conducted after a short storage period simulating a short transport/storage) from two weeks (non-active packages) to three weeks. Different package formats were studied (different sized trays and boxes, and even including alveoli trays), with “large box” format (≈10 kg fruit per box) showing the best results during this commercialization period. Particularly, the active large box better controlled the microbial growth of mandarins (with loads up to two log units lower after three weeks) and colour changes, while it induced the highest reductions of weight losses and the greatest acidity decrease of mandarins after three weeks (correspondent to the lowest maturity index changes among active samples), which was highly appreciated in the sensory analyses. This package format was then selected and validated at the industrial level during a long storage (up to 21 days) of mandarins. The active large box showed the same benefits, reducing microbial growth (together with decay incidence) and weight losses while maintaining the physicochemical quality (soluble solids, titratable acidity, firmness, and colour). In that sense, the shelf life of mandarins was extended from 14 days (non-active large box) to at least 21 days. As observed, the controlled release of EOs from the active large box extended the shelf life of mandarins either during a long cold transportation simulation or a commercialization period at non-recommended room temperature. 

## Figures and Tables

**Table 1:** Weight loss (%), soluble solid content (SSC; °Brix), titratable acidity (TA; %), BrimA maturity index (SSC-(3 × TA)), firmness (N) and colour index of mandarins packaged within different packages types (ST, small tray; SB, small box; LT, large tray; LT+, large tray with alveoli tray; and LB, large box), non-active (CT) and active, during a cold storage period (CS; 5 days at 8 °C) followed by a commercialization simulation (room temperature) up to 3 weeks (CS+1 wk, CS+2 wk and CS+3 wk) (n = 3 ± SD). Least significant differences are represented between parentheses.

	Package	Activity	Weight Loss	SSC	TA	BrimA	Firmness	Colour
Day 0			-	15.1 ± 0.4	1.23 ± 0.12	11.4 ± 0.2	19.3 ± 1.5	7.8 ± 0.5
CS	ST	CT	0.3 ± 0.1	12.5 ± 0.5	1.58 ± 0.12	7.8 ± 0.2	16.5 ± 1.0	7.6 ± 1.0
		Active	0.3 ± 0.1	14.4 ± 1.4	1.48 ± 0.10	9.9 ± 1.3	17.6 ± 2.5	8.3 ± 0.3
	SB	CT	1.5 ± 0.5	13.4 ± 0.9	1.67 ± 0.13	8.4 ± 1.2	19.4 ± 2.0	7.7 ± 1.0
		Active	1.1 ± 0.2	12.9 ± 0.8	1.63 ± 0.03	8.0 ± 0.7	19.9 ± 2.8	7.9 ± 0.3
	LT	CT	0.8 ± 0.2	12.5 ± 0.5	1.62 ± 0.21	7.7 ± 0.7	20.4 ± 1.9	8.6 ± 0.6
		Active	0.7 ± 0.2	13.0 ± 0.8	1.52 ± 0.11	8.5 ± 0.6	20.0 ± 3.4	8.3 ± 0.8
	LT+	CT	0.9 ± 0.2	13.6 ± 0.5	1.59 ± 0.11	8.8 ± 0.6	20.7 ± 2.8	8.2 ± 0.6
		Active	1.3 ± 0.3	13.4 ± 0.7	1.48 ± 0.19	9.0 ± 1.1	18.8 ± 1.6	8.2 ± 0.7
	LB	CT	0.1 ± 0.0	13.9 ± 1.7	1.45 ± 0.09	9.5 ± 1.8	19.9 ± 3.3	8.1 ± 0.8
		Active	0.1 ± 0.0	12.7 ± 1.1	1.67 ± 0.10	7.7 ± 1.0	19.7 ± 2.1	8.3 ± 0.8
CS+1 wk	ST	CT	1.7 ± 0.2	13.2 ± 1.2	0.97 ± 0.12	10.3 ± 1.4	19.1 ± 1.9	6.9 ± 0.8
		Active	1.8 ± 0.3	13.0 ± 0.8	1.11 ± 0.15	9.7 ± 0.5	18.9 ± 1.0	7.2 ± 0.5
	SB	CT	5.7 ± 0.5	13.4 ± 1.3	1.10 ± 0.09	10.1 ± 1.3	18.4 ± 1.8	7.1 ± 0.9
		Active	4.5 ± 0.3	13.3 ± 1.1	1.11 ± 0.15	10.0 ± 1.1	18.4 ± 2.9	7.8 ± 0.7
	LT	CT	2.9 ± 0.4	13.5 ± 0.8	1.15 ± 0.08	10.0 ± 0.7	19.6 ± 3.1	6.8 ± 0.7
		Active	3.1 ± 0.4	13.0 ± 0.6	1.04 ± 0.15	9.9 ± 0.8	18.1 ± 1.9	6.8 ± 0.7
	LT+	CT	4.0 ± 0.5	13.4 ± 1.2	1.00 ± 0.18	10.4 ± 0.9	20.5 ± 2.8	7.2 ± 0.8
		Active	4.6 ± 0.5	13.2 ± 1.0	1.02 ± 0.10	10.1 ± 0.8	18.5 ± 1.5	7.4 ± 0.4
	LB	CT	2.60.4	14.3 ± 1.0	1.06 ± 0.13	11.1 ± 0.8	18.0 ± 2.5	7.5 ± 1.0
		Active	1.6 ± 0.1	14.6 ± 1.0	1.02 ± 0.15	11.5 ± 1.0	18.6 ± 1.5	7.3 ± 0.9
CS+2 wk	ST	CT	4.5 ± 0.9	11.5 ± 1.1	0.84 ± 0.09	9.0 ± 0.9	15.7 ± 1.7	8.0 ± 0.6
		Active	5.1 ± 0.7	14.4 ± 0.8	1.08 ± 0.15	11.2 ± 0.9	16.7 ± 1.3	8.1 ± 0.6
	SB	CT	11.4 ± 0.8	14.6 ± 0.4	0.85 ± 0.10	12.0 ± 0.5	14.2 ± 1.1	8.8 ± 0.7
		Active	10.2 ± 0.2	12.9 ± 1.1	1.06 ± 0.09	9.7 ± 1.3	15.0 ± 1.4	8.4 ± 0.4
	LT	CT	11.7 ± 1.2	13.5 ± 0.5	1.24 ± 0.13	9.8 ± 0.7	17.5 ± 1.5	8.6 ± 1.0
		Active	7.4 ± 1.1	14.1 ± 0.8	1.18 ± 0.07	10.5 ± 0.8	17.2 ± 1.6	8.3 ± 1.1
	LT+	CT	8.1 ± 0.3	13.3 ± 0.6	0.93 ± 0.08	10.5 ± 0.7	17.3 ± 1.0	8.2 ± 0.3
		Active	8.5 ± 0.4	13.8 ± 0.8	0.98 ± 0.08	10.9 ± 0.8	14.8 ± 1.2	8.1 ± 0.5
	LB	CT	8.7 ± 1.3	14.5 ± 1.1	1.15 ± 0.15	11.0 ± 1.1	16.4 ± 1.4	8.6 ± 0.9
		Active	5.9 ± 0.5	13.9 ± 1.0	1.22 ± 0.15	10.2 ± 0.8	17.1 ± 1.4	8.7 ± 1.0
CS+3 wk	ST	CT	11.0 ± 1.5	14.5 ± 0.5	0.93 ± 0.07	11.7 ± 0.6	16.7 ± 1.7	8.1 ± 0.5
		Active	8.2 ± 0.1	13.7 ± 1.0	0.96 ± 0.07	10.8 ± 0.9	17.6 ± 1.5	8.2 ± 0.3
	SB	CT	14.0 ± 0.9	16.0 ± 1.2	1.01 ± 0.07	13.0 ± 1.3	14.8 ± 1.0	8.7 ± 0.7
		Active	12.9 ± 0.3	13.9 ± 0.4	0.87 ± 0.05	11.4 ± 0.5	14.8 ± 1.4	8.8 ± 1.0
	LT	CT	15.9 ± 0.7	14.4 ± 0.4	0.90 ± 0.05	11.7 ± 0.4	17.5 ± 1.5	8.7 ± 0.8
		Active	9.9 ± 1.2	14.1 ± 0.4	0.90 ± 0.11	11.4 ± 0.2	14.8 ± 1.0	8.2 ± 0.7
	LT+	CT	10.2 ± 0.9	15.7 ± 0.4	0.93 ± 0.08	12.9 ± 0.3	15.4 ± 1.6	8.3 ± 0.7
		Active	10.9 ± 0.7	15.5 ± 0.4	1.04 ± 0.09	12.4 ± 1.2	15.0 ± 1.4	8.2 ± 1.0
	LB	CT	14.2 ± 1.2	15.2 ± 1.5	1.12 ± 0.11	11.8 ± 1.3	15.8 ± 1.5	8.6 ± 0.6
		Active	9.0 ± 0.5	13.5 ± 0.9	0.85 ± 0.09	10.9 ± 1.0	15.9 ± 1.3	8.7 ± 1.0
Packaging type (A)			(0.7) ^‡^	(0.5) ^†^	(0.05) *	(0.4) *	(0.7) ^†^	(0.2) *
Package activity (B)			(0.4) ^‡^	ns	ns	ns	ns	ns
Storage time (C)			(0.6) ^‡^	(0.6) ^‡^	(0.08) ^‡^	(0.6) ^‡^	(0.9) ^‡^	(0.3) ^‡^
A × B			(1.2) ^‡^	(0.8) ^‡^	ns	(0.8) ^‡^	(1.2) ^‡^	ns
A × C			(1.4) ^‡^	(1.0) ^†^	(0.18) ^‡^	(1.0) ^†^	(1.9) ^‡^	(0.4) *
B × C			(0.9) ^‡^	(0.6) ^†^	(0.01) ^*^	(0.5) *	ns	ns
A × B × C			(2.0) ^‡^	(1.4) ^†^	(0.25) ^‡^	(1.9) ^‡^	ns	ns

ns: not significant (*p* > 0.05); *, ^†^ and ^‡^ significance for *p* ≤ 0.05, 0.01 and 0.001, respectively.

**Table 2:** Microbial loads (log CFU cm^−2^) of mandarins packaged within different packages types (ST, small tray; SB, small box; LT, large tray; LT+, large tray with alveoli tray; and LB, large box), non-active (CT) and active, during a cold storage period (CS; 5 days at 8 °C) followed by a commercialization simulation (room temperature) up to 3 weeks (CS+1 wk, CS+2 wk and CS+3 wk) (*n* = 3 ± SD). Least significant differences are represented between parentheses.

Storage	Package	Activity	Mesophiles	Psychrophiles	Enterobacteria	Yeast	Moulds
Day 0			1.3 ± 0.1	1.0 ± 0.1	<0.5	2.3 ± 0.5	2.2 ± 0.3
CS	ST	CT	1.9 ± 0.1	1.0 ± 0.7	0.5 ± 0.2	2.3 ± 0.4	1.9 ± 0.4
		Active	1.6 ± 0.2	0.6 ± 0.8	0.5 ± 0.3	2.3 ± 0.1	1.5 ± 0.3
	SB	CT	2.3 ± 0.1	1.9 ± 0.2	1.2 ± 0.1	2.0 ± 0.2	2.3 ± 0.1
		Active	1.8 ± 0.2	1.1 ± 0.2	<0.5	1.9 ± 0.3	1.8 ± 0.1
	LT	CT	2.5 ± 0.3	2.5 ± 0.3	1.6 ± 0.2	2.4 ± 0.1	2.3 ± 0.2
		Active	1.9 ± 0.2	0.8 ± 0.2	<0.5	1.9 ± 0.3	2.1 ± 0.2
	LT+	CT	2.3 ± 0.4	1.1 ± 0.4	0.5 ± 0.2	2.0 ± 0.2	2.0 ± 0.1
		Active	1.7 ± 0.3	0.5 ± 0.4	<0.5	1.5 ± 0.5	2.1 ± 0.1
	LB	CT	1.9 ± 0.4	2.1 ± 0.8	<0.5	2.2 ± 0.1	1.8 ± 0.2
		Active	1.2 ± 0.2	1.4 ± 0.2	<0.5	1.7 ± 0.4	1.7 ± 0.2
CS+1 wk	ST	CT	2.4 ± 0.3	2.0 ± 0.4	0.8 ± 0.1	2.4 ± 0.2	2.7 ± 0.1
		Active	2.0 ± 0.4	1.6 ± 0.2	0.5 ± 0.4	1.7 ± 0.3	2.4 ± 0.1
	SB	CT	2.6 ± 0.2	2.0 ± 0.6	1.5 ± 0.1	1.9 ± 0.3	2.7 ± 0.2
		Active	2.1 ± 0.4	1.3 ± 0.2	<0.5	1.7 ± 0.4	2.4 ± 0.2
	LT	CT	2.5 ± 0.2	2.3 ± 0.5	1.7 ± 0.1	2.0 ± 0.3	2.6 ± 0.2
		Active	1.7 ± 0.3	1.7 ± 0.2	0.5 ± 0.1	2.0 ± 0.1	2.6 ± 0.1
	LT+	CT	2.4 ± 0.2	1.2 ± 0.1	1.1 ± 0.4	2.3 ± 0.1	1.9 ± 0.2
		Active	2.0 ± 0.2	0.2 ± 0.1	<0.5	1.7 ± 0.1	1.8 ± 0.2
	LB	CT	2.3 ± 0.1	1.6 ± 0.6	0.8 ± 0.2	2.3 ± 0.1	2.5 ± 0.3
		Active	1.3 ± 0.3	0.6 ± 0.3	<0.5	2.0 ± 0.2	2.0 ± 0.2
CS+2 wk	ST	CT	2.5 ± 0.1	1.2 ± 0.1	1.3 ± 0.3	2.2 ± 0.1	3.0 ± 0.3
		Active	1.9 ± 0.1	1.1 ± 0.4	<0.5	1.9 ± 0.2	3.0 ± 0.2
	SB	CT	2.7 ± 0.3	2.1 ± 0.2	1.2 ± 0.2	2.0 ± 0.2	2.6 ± 0.2
		Active	1.9 ± 0.3	1.4 ± 0.2	0.5 ± 0.2	1.6 ± 0.4	2.2 ± 0.1
	LT	CT	2.3 ± 0.3	1.4 ± 0.2	1.2 ± 0.3	1.5 ± 0.5	2.5 ± 0.1
		Active	2.1 ± 0.3	1.5 ± 0.9	<0.5	1.5 ± 0.3	2.1 ± 0.2
	LT+	CT	2.1 ± 0.2	2.1 ± 0.5	1.2 ± 0.2	2.0 ± 0.2	2.7 ± 0.3
		Active	1.3 ± 0.4	1.4 ± 0.2	<0.5	1.3 ± 0.2	2.0 ± 0.3
	LB	CT	2.6 ± 0.3	1.6 ± 0.2	0.5 ± 0.4	2.3 ± 0.2	2.6 ± 0.1
		Active	1.6 ± 0.1	1.8 ± 0.2	0.6 ± 0.4	1.2 ± 0.1	2.2 ± 0.1
CS+3 wk	ST	CT	2.8 ± 0.3	2.2 ± 0.4	1.1 ± 0.2	2.2 ± 0.2	3.1 ± 0.2
		Active	1.9 ± 0.2	2.1 ± 0.3	0.9 ± 0.2	2.1 ± 0.1	2.3 ± 0.2
	SB	CT	2.3 ± 0.1	2.5 ± 0.2	1.6 ± 0.4	1.7 ± 0.2	2.0 ± 0.1
		Active	1.8 ± 0.2	1.5 ± 0.3	<0.5	1.2 ± 0.1	1.9 ± 0.1
	LT	CT	2.6 ± 0.3	2.6 ± 0.5	0.9 ± 0.2	2.0 ± 0.4	3.4 ± 0.2
		Active	1.8 ± 0.2	1.7 ± 0.1	<0.5	1.3 ± 0.2	2.4 ± 0.3
	LT+	CT	2.3 ± 0.2	1.9 ± 0.3	1.0 ± 0.1	1.8 ± 0.2	2.5 ± 0.2
		Active	1.7 ± 0.2	1.0 ± 0.1	<0.5	1.2 ± 0.1	1.9 ± 0.3
	LB	CT	2.6 ± 0.2	1.8 ± 0.4	<0.5	2.4 ± 0.2	3.5 ± 0.2
		Active	1.9 ± 0.2	1.3 ± 0.2	0.5 ± 0.3	2.0 ± 0.4	3.0 ± 0.3
Packaging type (A)			(0.2) ^‡^	(0.4) ^‡^	(0.2) ^‡^	(0.3) ^‡^	(0.2) ^‡^
Package activity (B)			(0.1) ^‡^	(0.2) ^‡^	(0.1) ^‡^	(0.2) ^‡^	(0.1) ^‡^
Storage time (C)			(0.2) ^‡^	(0.4) ^‡^	(0.2) ^‡^	(0.3) ^‡^	(0.2) ^‡^
A×B			ns	ns	(0.2) ^‡^	ns	ns
A×C			(0.5) ^‡^	(0.8) ^‡^	(0.4) ^‡^	(0.6) ^‡^	(0.4) ^‡^
B×C			(0.3) ^‡^	(0.5) ^‡^	(0.2) ^‡^	(0.4) ^‡^	(0.3) ^‡^
A×B×C			ns	ns	(0.5) ^‡^	ns	(0.6) ^‡^

ns: not significant (*p* > 0.05); ^‡^, significance for *p* ≤ 0.001.

## Supplementary Materials

The following supplementary materials are available online at https://www.mdpi.com/2304-8158/9/5/590/s1. Table S1: CIE colour parameters (*L*, *a*, *b*; Chroma, °Hue) of mandarins (Experiment 1), Table S2: CIE colour parameters (*L*, *a*, *b*; Chroma, °Hue) of mandarins (Experiment 2).
