# Supplementary material for: Active Cardboard Box with Smart Internal Lining Based on Encapsulated Essential Oils for Enhancing the Shelf Life of Fresh Mandarins
Source: Foods. 2020 May 6;9(5):590. doi: 10.3390/foods9050590 (PMC7278779; doi:10.3390/foods9050590)
Supplement: Supplementary file 1 [file foods-09-00590-s001.zip › Supplementary material 2.docx]

|  | Activity | L* | a* | b* |  | Chroma | ºHue |
| --- | --- | --- | --- | --- | --- | --- | --- |
| Day 0 |  | 64.1±0.7 | 30.0±1.3 | 61.6±1.3 |  | 68.5±1.5 | 64.0±0.9 |
|  |  |  |  |  |  |  |  |
| Day 7 | CT | 63.5±0.3 | 24.9±2.0 | 65.1±1.3 |  | 69.7±1.7 | 69.1±1.4 |
|  | Active | 63.9±0.7 | 29.1±1.9 | 67.7±2.0 |  | 73.7±1.5 | 66.8±1.8 |
| Day 14 | CT | 62.8±0.7 | 25.8±1.8 | 57.8±4.3 |  | 63.3±3.8 | 65.8±2.4 |
|  | Active | 63.7±0.8 | 21.7±2.1 | 64.7±2.7 |  | 68.2±2.6 | 71.4±1.9 |
| Day 21 | CT | 60.8±0.5 | 21.0±2.1 | 40.5±1.1 |  | 45.5±1.2 | 62.6±2.6 |
|  | Active | 61.0±0.9 | 24.4±2.4 | 42.9±2.8 |  | 49.4±1.9 | 60.3±3.6 |
|  |  |  |  |  |  |  |  |
| Packaging activity (A) | | ns | (2.3)† | ns |  | (2.8)‡ | ns |
| Storage time (B) | | (1.3)‡ | (4.4)‡ | (1.4)‡ |  | (4.0)‡ | (3.8)‡ |
| A×B | | ns | ns | (1.5)† |  | ns | (4.1)† |

ns: not significant (p>0.05); † and ‡ significance for p≤ 0.01 and 0.001, respectively.
