# Supplementary material for: Active Cardboard Box with Smart Internal Lining Based on Encapsulated Essential Oils for Enhancing the Shelf Life of Fresh Mandarins
Source: Foods. 2020 May 6;9(5):590. doi: 10.3390/foods9050590 (PMC7278779; doi:10.3390/foods9050590)
Supplement: Supplementary file 1 [file foods-09-00590-s001.zip › Supplementary material 1.docx]

|  | Type | Activity | L* | a* | b* |  | Chroma | ºHue |
| --- | --- | --- | --- | --- | --- | --- | --- | --- |
| Day 0 | | | 59.5±1.0 | 28.6±2.2 | 61.2±2.4 |  | 67.6±2.7 | 65.0±1.6 |
|  |  |  |  |  |  |  |  |  |
| CS | ST | CT | 61.4±1.0 | 28.0±1.6 | 60.1±1.8 |  | 66.3±1.6 | 65.0±2.6 |
|  |  | Active | 61.0±1.2 | 30.5±1.4 | 60.0±2.1 |  | 67.3±2.2 | 63.0±1.0 |
|  | SB | CT | 62.3±1.6 | 28.2±3.6 | 58.8±1.6 |  | 65.3±2.7 | 64.4±2.5 |
|  |  | Active | 61.6±1.1 | 29.7±0.7 | 61.0±2.3 |  | 67.8±2.2 | 64.0±0.7 |
|  | LT | CT | 62.3±0.9 | 31.1±1.2 | 58.2±2.7 |  | 66.0±2.4 | 61.9±1.5 |
|  |  | Active | 62.8±1.0 | 30.0±2.4 | 57.5±1.8 |  | 64.9±1.8 | 62.4±2.1 |
|  | LT+ | CT | 63.3±0.9 | 29.5±1.8 | 56.5±1.2 |  | 63.8±1.6 | 62.5±1.3 |
|  |  | Active | 63.3±1.3 | 29.9±2.0 | 57.8±2.2 |  | 65.1±2.2 | 62.7±1.7 |
|  | LB | CT | 62.8±1.4 | 29.7±2.3 | 58.5±2.5 |  | 65.9±2.5 | 63.2±2.0 |
|  |  | Active | 62.7±1.3 | 30.6±2.2 | 58.7±2.7 |  | 66.2±2.4 | 62.5±2.2 |
|  |  |  |  |  |  |  |  |  |
| CS+1 wk | ST | CT | 66.1±1.6 | 28.4±1.6 | 62.3±1.9 |  | 68.5±1.6 | 65.5±2.1 |
|  |  | Active | 66.1±1.4 | 29.5±1.1 | 62.4±2.2 |  | 69.0±1.8 | 64.7±1.4 |
|  | SB | CT | 66.4±2.4 | 28.9±3.1 | 61.2±1.8 |  | 67.7±1.5 | 64.8±2.8 |
|  |  | Active | 65.7±1.7 | 30.6±1.8 | 59.9±3.1 |  | 67.3±2.6 | 62.9±2.1 |
|  | LT | CT | 66.3±1.2 | 28.5±1.4 | 63.4±2.4 |  | 69.5±1.8 | 65.7±1.8 |
|  |  | Active | 66.4±1.2 | 28.6±2.0 | 63.6±1.5 |  | 69.7±0.8 | 65.8±1.9 |
|  | LT+ | CT | 66.1±2.0 | 29.2±2.0 | 61.6±3.0 |  | 68.2±2.4 | 64.6±2.2 |
|  |  | Active | 65.6±1.4 | 29.9±1.4 | 61.8±1.2 |  | 68.6±1.4 | 64.2±1.1 |
|  | LB | CT | 64.9±1.4 | 30.1±2.7 | 61.8±2.6 |  | 68.8±1.8 | 64.0±2.7 |
|  |  | Active | 65.2±1.2 | 29.1±2.4 | 61.8±1.8 |  | 68.4±1.9 | 64.8±2.4 |
|  |  |  |  |  |  |  |  |  |
| CS+2 wk | ST | CT | 62.5±1.2 | 28.9±1.6 | 57.8±1.6 |  | 64.1±1.1 | 63.1±1.3 |
|  |  | Active | 62.7±1.2 | 28.9±1.4 | 57.3±1.6 |  | 59.4±3.8 | 61.8±2.7 |
|  | SB | CT | 61.1±1.0 | 30.5±2.1 | 57.1±1.2 |  | 63.7±2.8 | 61.2±2.0 |
|  |  | Active | 62.8±0.9 | 30.2±1.1 | 57.7±1.0 |  | 64.8±1.1 | 62.5±1.6 |
|  | LT | CT | 61.7±0.9 | 30.4±2.3 | 57.5±1.9 |  | 64.8±0.8 | 61.8±2.6 |
|  |  | Active | 62.5±1.9 | 29.8±2.2 | 58.1±2.3 |  | 65.3±1.5 | 62.8±2.5 |
|  | LT+ | CT | 61.8±1.1 | 29.0±1.3 | 57.3±2.5 |  | 59.4±1.3 | 60.7±2.0 |
|  |  | Active | 63.0±0.7 | 29.6±1.5 | 58.3±0.7 |  | 65.4±0.9 | 63.1±1.4 |
|  | LB | CT | 61.7±0.7 | 29.8±1.4 | 56.8±3.2 |  | 60.7±2.6 | 60.7±2.0 |
|  |  | Active | 62.4±1.4 | 30.9±2.1 | 57.3±1.8 |  | 65.4±1.2 | 62.0±2.6 |
|  |  |  |  |  |  |  |  |  |
| CS+3 wk | ST | CT | 62.6±0.8 | 29.3±1.5 | 58.1±1.4 |  | 65.3±1.3 | 62.0±1.7 |
|  |  | Active | 62.5±0.4 | 29.7±0.8 | 57.8±0.8 |  | 65.6±1.1 | 62.9±1.7 |
|  | SB | CT | 62.0±1.0 | 31.2±1.7 | 58.1±2.0 |  | 65.8±1.8 | 61.6±1.7 |
|  |  | Active | 61.6±1.5 | 31.0±2.0 | 57.6±1.7 |  | 65.1±0.9 | 61.9±2.2 |
|  | LT | CT | 62.2±1.6 | 30.8±1.3 | 57.2±1.7 |  | 65.1±1.6 | 63.3±1.2 |
|  |  | Active | 62.5±1.0 | 29.7±1.5 | 58.4±1.6 |  | 64.9±0.8 | 62.8±1.1 |
|  | LT+ | CT | 62.7±1.4 | 30.4±1.7 | 58.7±2.1 |  | 65.8±1.5 | 62.9±1.9 |
|  |  | Active | 62.4±1.2 | 30.0±2.6 | 58.6±1.8 |  | 64.7±1.2 | 62.5±2.3 |
|  | LB | CT | 62.2±0.7 | 31.2±1.2 | 58.2±1.3 |  | 66.0±0.8 | 61.8±1.5 |
|  |  | Active | 62.1±1.4 | 31.0±2.0 | 57.7±2.0 |  | 65.6±1.2 | 61.7±2.2 |
|  |  |  |  |  |  |  |  |  |
| Packaging format (A) | | | (0.3)* | (0.3)* | ns |  | ns | (0.7)† |
| Package activity (B) | | | ns | ns | ns |  | ns | ns |
| Storage time (C) | | | (0.6)‡ | (0.9)‡ | (1.0)‡ |  | (0.9)‡ | (0.7)† |
| A×B | | | ns | ns | ns |  | ns | ns |
| A×C | | | (1.3)‡ | ns | (2.2)‡ |  | (1.6)† | (1.5)† |
| B×C | | | ns | ns | ns |  | ns | ns |
| A×B×C | | | ns | ns | ns |  | ns | ns |

ns: not significant (p>0.05); *, † and ‡ significance for p≤0.05, 0.01 and 0.001, respectively.
